# Plants synthesize ergothioneine, showing a link to abiotic stress

**DOI:** 10.1111/plb.70218

**Published:** 2026-04-28

**Authors:** C. Kock, N. Gutsche, S. Walter, S. Zachgo

**Affiliations:** ^1^ Division of Botany Osnabrück University Osnabrück Germany; ^2^ Facility for Mass Spectrometry Osnabrück University Osnabrück Germany

**Keywords:** Abiotic stress, antioxidant, EGT biosynthesis, ergothioneine, plant metabolism, Viridiplantae

## Abstract

- Ergothioneine (EGT) is a sulphur‐containing histidine derivative and a potent antioxidant that exhibits beneficial effects on human health. Thus far, only fungi and certain bacteria have been reported to produce EGT, whereas plants are assumed to rely on an uptake of EGT. Here, the presence of EGT biosynthetic genes and their functionality were investigated in Viridiplantae.
- The biosynthetic genes *EGT1* and *EGT2* from yeast were used for transcriptome and genome analyses in evolutionarily informative species across Viridiplantae. Targeted metabolomics (HPLC‐MRM/MS) was used to quantify EGT in selected algae and land plants grown under control conditions and exposed to abiotic stress.
- *EGT1* and *EGT2* genes were identified in streptophyte algae, bryophytes, lycophytes, monilophytes, and gymnosperms. Targeted metabolomic profiling demonstrated endogenous EGT production in diverse algae and land plants, refuting the long‐standing view that plants cannot synthesize this antioxidant. Notably, *EGT1* genes do not exist in angiosperms, which likely lost this gene and the capability to synthesize EGT. After high light and heat stress exposure, EGT synthesis increases significantly in the streptophyte algae *Klebsormidium nitens* and the moss *Physcomitrium patens*, suggesting that EGT also exerts an antioxidant function in plants.
- Contrary to previous assumptions, various plants possess *EGT* genes and are capable of synthesizing EGT. Abiotic stress experiments reveal a link between EGT and the plant stress response, opening new avenues for research in stress signalling and adaptation – areas that are also relevant for enhancing crop resilience and nutritional quality.

## Graphical abstract

Various plants possess ergothioneine biosynthetic genes and can synthesize this antioxidant, where increased EGT levels under abiotic stress conditions indicate protective functions.

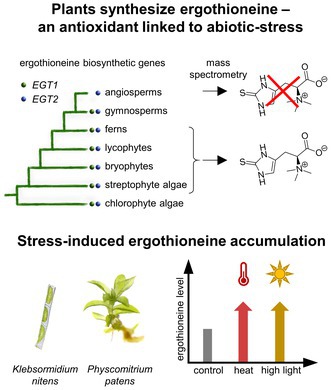

## INTRODUCTION

Ergothioneine (EGT) is a sulfur‐containing derivative of the amino acid histidine that was first isolated a century ago from the ergot of rye (*Claviceps purpurea*), hence the name ergothioneine. EGT is attracting substantial research interest due to its antioxidant capacity and diverse benefits for human health (Cheah & Halliwell 2021; Chen *et al*. 2024). According to the literature, EGT originates exclusively from microbial and fungal synthesis and is thought to be taken up from the environment by plants and animals (Park *et al*. 2010; Ames 2018).

While highly abundant low‐molecular‐weight thiols such as glutathione (GSH) provide a crucial buffer for redox homeostasis, EGT displays distinct physicochemical properties (Stoffels *et al*. 2017). EGT is chemically and thermally more stable and exhibits lower reactivity and greater resistance to autooxidation than GSH (Hartman 1990). In addition, EGT scavenges singlet oxygen more efficiently than GSH and can chelate divalent cations such as Cu^2+^, counteracting DNA‐damaging properties (Zhu *et al*. 2011; Stoffels *et al*. 2017). Human studies suggest that GSH acts as the main cellular thiol buffer and EGT likely functions as a secondary antioxidant that becomes important when the redox equilibrium is disturbed or when primary antioxidant reserves are depleted (Cheah & Halliwell 2021; Paul 2022). In humans, decrease of EGT blood levels leads to increased oxidative stress and frailty. Numerous studies have demonstrated that EGT plays a broad role in preventing a variety of mammalian diseases, including neurodegenerative disorders such as Parkinson's and Alzheimer's disease. The administration of EGT has been associated with improved health outcomes, longer lifespans, and a reduction in the risk and progression of oxidative stress‐related diseases (Paul & Snyder 2010; Kameda *et al*. 2020; Cheah & Halliwell 2021; Wu *et al*. 2021; Paul 2022; Petrovic *et al*. 2025).

In plants, the functions of EGT remain largely unexplored. EGT accumulation has been reported in association with mycorrhizal symbiosis and under altered cultivation regimes. Moreover, exogenous application of EGT enhanced flower formation and seed yield in *Arabidopsis thaliana* (Park *et al*. 2010; Carrara *et al*. 2023; Koshiyama *et al*. 2024; Das *et al*. 2026).

The fungal EGT biosynthetic pathway utilizes histidine and cysteine as substrates and is encoded by the *EGT1* and *EGT2* genes. The bifunctional EGT1 enzyme catalyses the triple methylation of histidine to hercynine and subsequent conjugation with cysteine and oxygen forming hercynylcysteine sulfoxide. EGT2 then performs the final step, a C‐S bond cleavage reaction, thereby producing EGT (Pluskal *et al*. 2014). In bacteria, instead of a single *EGT1* gene, two separate single‐domain genes together provide the same catalytic activities. Although the pathway of EGT production is conserved in fungi and bacteria (Jones *et al*. 2014), it remains undocumented in plants, where supply is assumed to occur via uptake of exogenous EGT, synthesized by fungi and/or bacteria. Here, we report the identification of *EGT* biosynthetic genes and the detection of EGT production in diverse plant lineages. Furthermore, selected plants were exposed to abiotic stress conditions and the detected increase in EGT levels suggests a role for this antioxidant in the stress response.

## MATERIALS AND METHODS

### Cultivation of investigated organisms

*Schizosaccharomyces pombe* was cultivated in minimal synthetic defined base (Clontech Laboratories, Inc., Mountain View, USA) liquid medium for 48 h at 30 °C and 180 rpm. All plants were cultivated under long‐day conditions (16 h light/8 h dark) at 22 °C. Light intensity was maintained at 40 μmol m^−2^ s^−1^ for *Chara braunii* (NIES‐1604) and *Chlamydomonas reinhardtii* (strain CC‐124) or 80 μmol m^−2^ s^−1^ for *Klebsormidium nitens* (NIES‐2285), *Spirogyra pratensis* (MZCH 10213), *Marchantia polymorpha* ssp. *ruderalis* (BoGa, Bot. Garden, University of Osnabrück), *Anthoceros agrestis* (BONN), *Physcomitrium patens* (Reute), *Ceratopteris richardii* (strain Hn‐n), *Arabidopsis thaliana* (Col‐0), and *Thuja plicata* (Bot. Garden, University of Osnabrück). Organisms were grown in the following media: *Chlamydomonas reinhardtii* in TAP liquid medium (Gorman & Levine 1965), *Klebsormidium nitens* and *Spirogyra pratensis* in liquid C‐Medium (Provasoli 1959), *Marchantia polymorpha*, *Ceratopteris richardii* and *Anthoceros agrestis* on ½ Gamborg B5 medium with vitamins (Duchefa, Netherlands) + 1.4% Phyto‐Agar (Duchefa, Netherlands), *Physcomitrium patens* on KNOP‐ME medium (Reski & Abel 1985) + 1.2% Phyto‐Agar, *Arabidopsis thaliana* on Murashige and Skoog medium (Duchefa, Netherlands) + 1% Phyto‐Agar. *Chara braunii* was cultivated in sterilized water supplemented with plant care aquarium soil (Tropica, Denmark).

### Sterilization and cultivation of *Thuja plicata*

*Thuja plicata* branch buds were harvested late February and sterilized with 70% ethanol and 0.01% Tween 20 for 10 min. Sterilized buds were cultivated on ½ Gamborg B5 medium with vitamins, 2 mg L^−1^ 2,4‐dichlorophenoxyacetic acid (2,4D), 0.5 mg L^−1^ 6‐Benzylaminopurine (BAP), 2% sucrose and 1% Phyto‐Agar for 2 months. The proliferating sterile callus tissue was harvested for mass spectrometry analyses.

### Stress treatment

*P. patens*, *K. nitens* and *S. pratensis* were selected for stress treatments and pre‐cultivated for 14 days under standard long‐day conditions, followed by 72 h stress treatments (cold stress: 12 °C, 80 μmol m^−2^ s^−1^; heat stress: 29 °C and 80 μmol m^−2^ s^−1^; high light: 22 °C and 300 μmol m^−2^ s^−1^).

### Bioinformatics and sequence analysis

Homology searches were conducted with the *Schizosaccharomyces pombe* EGT1 and EGT2 protein sequences as queries against publicly accessible genome and transcriptome repositories (see Dataset S1 for details), using blastp or tblastn with default settings. *EGT* biosynthetic genes were characterized by the presence of the S‐adenosyl‐L‐methionine (SAM)‐dependent methyltransferase (IPR019257) and the formylglycine‐generating enzyme (FGE)‐sulfatase (IPR005532) domains in this order within EGT1, and a pyridoxal phosphate (PLP)‐binding L‐cysteine desulfhydrase (PTHR43092) in EGT2, as determined by InterProScan (version 106.0) analysis of the respective amino acid sequences (Blum *et al*. 2025).

### EGT isolation from yeast and whole plants

*S. pombe* cell pellets and plant tissue (50–100 mg fresh weight) were homogenized in 400 μL of 40% methanol containing 500 ng mL^−1^ of deuterium‐labelled ergothioneine (EGT‐D9, MW: 238.3 Da) as an internal standard. Homogenization was performed using two metal beads per 2 mL microcentrifuge tube in a BeadRuptor24 (Omni International, Kennesaw, USA). The resulting lysate was centrifuged at 13,000 g for 10 min to remove cell debris. Thereafter, 300 μL of the supernatant was transferred to a fresh microcentrifuge tube and further purified by centrifugation at 13,000 g for 30 min. Finally, 50 μL of the resulting supernatant was used for mass spectrometry analysis. L‐(+)‐ergothioneine (E7521) was purchased from Sigma‐Aldrich (St. Louis, MO, USA) and L‐(+)‐ergothioneine‐D9 (TRC‐E600003) was purchased from Toronto Research Chemicals, Inc. (North York, Toronto, Canada).

### Identification of EGT by mass spectrometry

Identification of EGT in yeast and plant extracts was achieved utilizing a high‐performance liquid chromatography coupled with multiple reaction monitoring mass spectrometry (HPLC‐MRM/MS). Samples were analysed using a Shimadzu LC‐20ADXR HPLC system (Shimadzu Corporation, Kyoto, Japan). Analytes were resolved on a C18 column (150 mm × 2.1 mm, 3 μm particle size, 100 Å pore size) employing a binary gradient mobile phase consisting of 0.1% formic acid in water (phase A) and 0.1% formic acid in 80% acetonitrile (phase B). This gradient commenced at 1% B for 0.30 min, and increased linearly to 90% B by 2.00 min. A rapid increase to 100% B was applied at 2.10 min and maintained for 1.00 min, before returning to initial conditions (1% B) within 0.10 min, with the total run time terminating at 5.00 min. Maintaining a consistent flow rate of 0.400 mL/min and column temperature of 40 °C ensured reproducible retention times and peak shapes. An injection volume of 1.00 μL was used for each analysis.

Following chromatographic separation, EGT and EGT‐D9 were detected and quantified using a SCIEX Triple Quad™ 5500+ (Marlborough, MA, USA) mass spectrometer operating in positive ion mode with a TurboSpray ionization source. Multiple reaction monitoring (MRM) transitions were optimized for both EGT and EGT‐D9 to ensure specificity and sensitivity. Specifically, transitions of m/z 230.081 → 127.000 (CE 29.0, CXP 6.0, DP 71.0) and 230.081 → 186.200 (CE 15.0, CXP 6.0, DP 71.0) were monitored for native EGT, while m/z 239.124 → 127.000 (CE 31.0, CXP 6.0, DP 76.0) and 239.124 → 195.200 (CE 17.0, CXP 6.0, DP 76.0) were observed for EGT‐D9. Parameters were optimized for maximal ionization efficiency, setting collision gas to low, curtain gas to 40.0, gas 1 and gas 2 to 70.0 and 60.0, respectively, and the ion source voltage to 3,200 V, while maintaining a source temperature of 525 °C.

### Quantification of EGT

Quantification of EGT was conducted as described by (Dumitrescu *et al*. 2022), adding monobromobimane (mBBr, 50 μg mL^−1^) to the isolation buffer. The resulting adduct elutes at 162 s with m/z 420.200 → 126.018 (CE 62.0, CXP 6.0, DP 150.0) (mBBr‐EGT) and 420.200 → 185.119 (CE 36.0, CXP 6.0, DP 150.0); the mBBr‐EGT‐D9 adduct shows m/z 429.271 → 126.029 (CE 61.0, CXP 10.0, DP 150.0) and 429.271 → 194.178 (CE 38.0, CXP 10.0, DP 150.0). A linear calibration curve (*R* ^2^ = 0.9994) spanning 1–729 ng mL^−1^ was used to calculate mBBr‐EGT concentrations (μg g^−1^ fresh weight) (Fig. S2). The fresh weights of the plant samples were measured before isolation for normalization.

### Statistical evaluation

The statistical analysis of data obtained from this study was performed by PRISM 10 (GraphPad Software, www.graphpad.com, San Diego, USA). Mean values were evaluated using analysis of variance (ANOVA), followed by a post hoc Tukey's test. Statistical significance was denoted by different lowercase letters (*P* < 0.05). Data visualization was executed with GraphPadPrism 10.

## RESULTS AND DISCUSSION

### Phylogenetic distribution of *EGT* biosynthetic genes

**Fig. 1:**
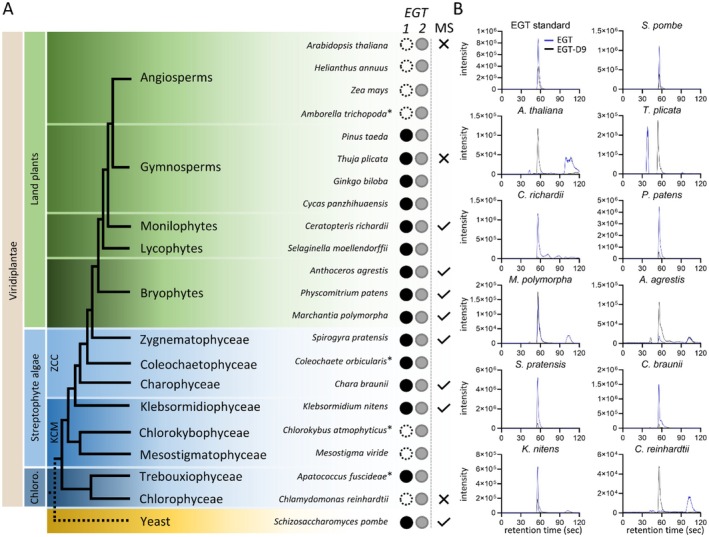
Discovery of *EGT1* and *EGT2* genes and endogenous EGT synthesis in Viridiplantae. (A) The cladogram depicts the presence of *EGT1* and *EGT2* genes across a selection of evolutionarily informative plant lineages using yeast as an outgroup. Whole genome or transcriptome (*) datasets were searched. Abbreviation: Chloro.: chlorophyte algae; KCM: Klebsormidiophyceae, Chlorokybophyceae, Mesostigmatophyceae; ZCC: Zygnematophyceae, Coleochaetophyceae, Charophyceae. Filled circles indicate identification of *EGT1/2* homologues, while circles with a dotted outline illustrate that no homologues were detected. EGT production was examined by mass spectrometry of sterile cultures from selected species; check marks (✓) denote detection of EGT and crosses (✕) absence of EGT. (B) HPLC‐MRM/MS extracted ion chromatograms show the separation and detection of endogenous EGT (blue line) and the deuterium‐labelled EGT‐D9 internal standard (black line), both exhibiting a retention time of 56.1 s. Y‐axis represents the intensity counts and X‐axis the retention time in seconds.

To identify homologues genes of the EGT biosynthetic pathway in Viridiplantae, publicly available genome and transcriptome datasets were interrogated that span the diversity of the green lineage. For a balanced sampling strategy, at least one representative from each major Viridiplantae clade was included in the analysis. The *Schizosaccharomyces pombe* protein sequences of EGT1 (SPBC1604.01) and EGT2 (SPBC660.12c) served as query sequences for the BLAST searches (Fig. 1A; Dataset S1).

EGT biosynthetic genes were detected in Viridiplantae. Homologues of *EGT2* were identified in all analysed plant species, and the presence of *EGT1* homologues was variable. A deep split separates Viridiplantae into chlorophytes and streptophytes, the latter comprise the paraphyletic streptophyte algae and the monophyletic land plants (de Vries & Archibald 2018). In chlorophytes, *Apatococcus fuscideae* (Trebouxiophyceae) possesses *EGT1*, whereas *EGT1* is absent in *Chlamydomonas reinhardtii* (Chlorophyceae). Streptophyte algae are subdivided into the KCM grade (Klebsormidiophyceae, Chlorokybophyceae and Mesostigmatophyceae) and the ZCC grade (Zygnematophyceae, Coleochaetophyceae, and Charophyceae) (de Vries *et al*. 2016). *EGT1* was identified in one representative of the KCM grade (*Klebsormidium nitens*) and in all analysed ZCC grade algae, including the Zygnematophyceae, which are considered the closest algal relatives of land plants (de Vries *et al*. 2016). Further analyses of additional algal lineages can elucidate the variable presence of *EGT1* among chlorophytes and within the KCM grade. Notably, *EGT1* homologues exist in all examined bryophyte, lycophyte, monilophyte and gymnosperm species but no *EGT1* homologues were identified in angiosperms. Representative angiosperms included species from eudicots and monocots as well as *Amborella*, a sister lineage to all other flowering plants (Zuntini *et al*. 2024). This distribution suggests that *EGT1* was lost in the most recent common ancestor of angiosperms. The identification of the *EGT1* and *EGT2* genes in Viridiplantae raises the question of whether plants can synthesize EGT *de novo*.

### Analysis of EGT synthesis in Viridiplantae

For targeted HPLC multiple reaction monitoring mass spectrometry (HPLC‐MRM/MS) of EGT, we selected evolutionary informative species for which sterile cultures were available or could be generated, to prevent uptake of external EGT (Fig. 1). The fission yeast *Schizosaccharomyces pombe* was used as a positive control and validation of the EGT isolation procedure. Calibration curves for EGT and the deuterium‐labelled EGT‐D9 internal standard using mass spectrometry showed retention times of 56.1 s. EGT and EGT‐D9 displayed mass‐to‐charge ratios (*m/z*) of 230.081 and 239.124, respectively, and enabled together with the specific MRM transitions the accurate identification of EGT in planta (Fig. 1B).

EGT was detected only in plants that possess both the *EGT1* and *EGT2* biosynthetic genes (Fig. 1B). Although the gymnosperm *Thuja plicata* harbours both genes, no EGT was measured, which may reflect tissue‐specific expression of the genes or limitations of the callus‐culture system used for the analysis. The absence of EGT production in angiosperms that lack *EGT1* supports the notion that flowering plants depend on external uptake of this compound, possibly via a transporter analogous to the human OCTN1 carrier (Gründemann *et al*. 2022). Collectively, our phylogenetic and metabolomic data demonstrate that plants synthesize EGT, overturning the long‐standing view that EGT biosynthesis is restricted to bacteria and fungi.

### EGT synthesis responds to abiotic stress

Biochemical studies in bacteria, fungi and mammalia showed the antioxidative properties of EGT (Bello *et al*. 2012; Pluskal *et al*. 2014; Petrovic *et al*. 2025). To address the role of EGT in plants, we investigated the transcriptional response of *EGT1* and *EGT2* to abiotic stress. Analysis of recently published transcriptome datasets (Goldbecker *et al*. 2025; Rieseberg *et al*. 2025; Sanchez‐Vera *et al*. 2026) revealed a deregulation of *EGT1* and *EGT2* expression under high light, heat and cold stress in the moss *Physcomitrium patens* and the alga *Spirogyra pratensis* (Fig. S1). We quantified EGT in these species grown under control conditions and after abiotic stress treatments using targeted mass spectrometry. Additionally, to broaden the evolutionary scope of this analysis, the alga *Klebsormidium nitens* was included. None of these treatments led to obvious phenotypic changes in any of these species (data not shown).

**Fig. 2:**
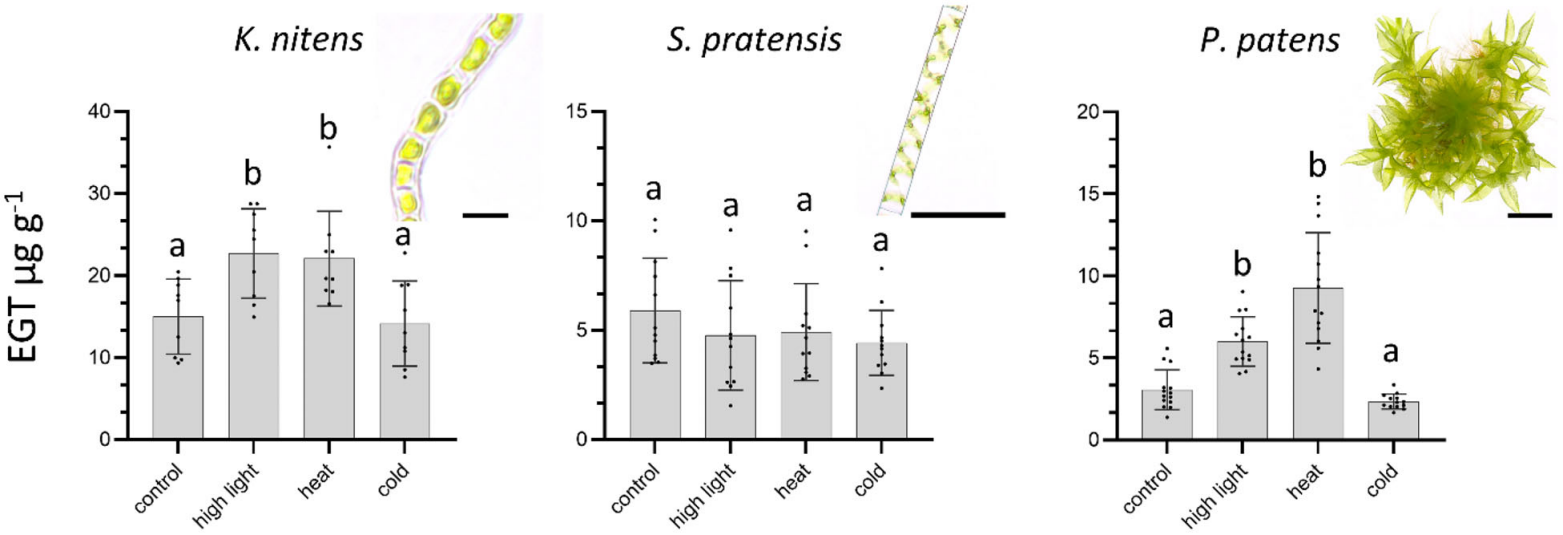
Analysis of the EGT response to abiotic stress conditions in *Klebsormidium nitens*, *Spirogyra pratensis* and *Physcomitrium patens*. Quantification of EGT (μg g^−1^ fresh weight plant material) in *K. nitens*, *S. pratensis* and *P. patens* after 14 days pre‐cultivation (80 μmol m^−2^ s^−1^) and subsequent exposure to high light (300 μmol m^−2^ s^−1^), heat (29 °C) or cold (12 °C) for 72 h. n ≥ 9, letters indicate statistically significant results. Data are represented as mean ± standard deviation. Statistical significance was tested using a one‐way ANOVA, followed by a Tukey's post hoc test (*P* < 0.05). Scale bars: *K. nitens* 10 μm, *S. pratensis* 100 μm, *P. patens* 1 mm.

*K. nitens* contained 15.04 ± 4.32 μg g^*−*1^ EGT in the control and showed a significant increase under high light and heat conditions, whereas exposure to cold revealed no effect (Fig. 2). For *S. pratensis*, 5.91 ± 2.28 μg g^−1^ EGT was measured in the control, and none of the stress treatments induced a significant change. In *P. patens*, the EGT concentration in control plants was 3.01 ± 1.18 μg g^*−*1^. Similarly to *K. nitens*, this moss showed a significant increase in EGT under high light and heat conditions, indicating species‐dependent responses of EGT levels to certain abiotic stresses. The moss *P. patens* and the terrestrial streptophyte alga *K. nitens* can tolerate the harsh and variable conditions encountered on land (Rensing *et al*. 2008; Hori *et al*. 2014). In contrast, the semi‐aquatic streptophyte alga *S. pratensis* predominately survives stress conditions by entering a dormant zygospore stage (Permann *et al*. 2022). The EGT response to abiotic stresses observed in *P. patens* and *K. nitens* may therefore contribute to their successful adaptation to terrestrial challenges. Together, these observations link the acquisition of the EGT biosynthetic pathway with adaptations to a terrestrial lifestyle.

Interestingly, the aquatic chlorophyte alga *Chlamydomonas reinhardtii* lacks an *EGT1* homologue and does not synthesize EGT, whereas the terrestrial chlorophyte alga *Apatococcus fuscideae* (Zahradníková *et al*. 2017) possesses an *EGT1* homologue and can be used to investigate its ability to synthesize EGT and a potential stress connection in Chlorophtya.

## CONCLUSION

The identification of a functional EGT biosynthetic pathway in plants provides a solid foundation for future studies on the physiological roles and antioxidant activity of EGT in Viridiplantae. Our results indicate that this unusual amino acid contributes to stress resistance, and further biochemical and genetic investigations can elucidate its protective roles and interactions with other crucial antioxidant systems such as GSH and ascorbate. Because dietary EGT accumulates in human tissues and has been associated with reduced disease risk, future work can define optimal concentrations of this antioxidant for plant growth as well as for human nutrition (Beelman *et al*. 2022). Understanding the function of EGT is especially relevant under rapidly changing environmental conditions, where an improved antioxidant capacity can enhance stress resistance and thus secure plant growth and crop yield.

## Author Contributions

CK and SZ designed the research. CK, NG and SW performed the research, and all authors analysed the data. CK and SZ wrote the manuscript with support from all co‐authors, and all authors reviewed the manuscript.

## Supporting information


**Dataset S1.** List of all identified *EGT1/2* homologues genes.

**Fig. S1.** Analysis of stress‐induced changes in *EGT1* and *EGT2* mRNA expression. *EGT1* and *EGT2* transcript levels were compared under diverse abiotic stress conditions in *Physcomitrium patens* and *Spirogyra pratensis*. (A) Expression data for *P. patens* were obtained from the Streptotime web interface (rshiny.gwdg.de/apps/streptotime/; Rieseberg *et al*. 2025) and represent responses to heat, cold and high light stress. *EGT1* (Pp3c20_13320V3.1) and *EGT2* (Pp3c8_13110V3.1) expression levels are displayed as box plots showing Z‐score transformed normalized log2 counts per million (CPM), obtained from the web interface. (B) For *S. pratensis*, *EGT1* (Sp_v1_0036840.1) and *EGT2* (Sp_v1_0018550.1) expression data originate from experiments utilizing temperature and light intensity gradients (peatmoss.plantcode.cup.uni‐freiburg.de/easy_gdb/index.php; Goldbecker *et al*. 2025) and are visualized as heatmaps, where red indicates high expression and yellow indicates low expression.
**Fig. S2**. Calibration curve for monobromobimane (mBBr)‐EGT quantification. Standards covering 1–729 ng mL^−1^ were prepared. For each standard the mBBr‐EGT chromatographic signal was integrated to obtain the peak area, and resulting data were used to generate the calibration equation.

## Data Availability

The data that supports the findings of this study are available in the supplementary material of this article.
