# Supplementary material for: Plants synthesize ergothioneine, showing a link to abiotic stress
Source: Plant Biol (Stuttg). 2026 Apr 28;28(5):1343–8. doi: 10.1111/plb.70218 (PMC13358676; doi:10.1111/plb.70218)
Supplement: Supplementary file 2 — Fig. S1. Analysis of stress‐induced changes in EGT1 and EGT2 mRNA expression. EGT1 and EGT2 transcript levels were compared under diverse abiotic stress conditions in Physcomitrium patens and Spirogyra pratensis. (A) Expression data for P. patens were obtained from the Streptotime web interface (rshiny.gwdg.de/apps/streptotime/; Rieseberg et al. 2025) and represent responses to heat, cold and high light stress. EGT1 (Pp3c20_13320V3.1) and EGT2 (Pp3c8_13110V3.1) expression levels are displayed as box plots showing Z‐score transformed normalized log2 counts per million (CPM), obtained from the web interface. (B) For S. pratensis, EGT1 (Sp_v1_0036840.1) and EGT2 (Sp_v1_0018550.1) expression data originate from experiments utilizing temperature and light intensity gradients (peatmoss.plantcode.cup.uni‐freiburg.de/easy_gdb/index.php; Goldbecker et al. 2025) and are visualized as heatmaps, where red indicates high expression and yellow indicates low expression. Fig. S2. Calibration curve for monobromobimane (mBBr)‐EGT quantification. Standards covering 1–729 ng mL−1 were prepared. For each standard the mBBr‐EGT chromatographic signal was integrated to obtain the peak area, and resulting data were used to generate the calibration equation. [file PLB-28-1343-s002.docx]

**Supporting Figure 1:** Analysis of stress‑induced changes in *EGT1* and *EGT2* mRNA expression. *EGT1* and *EGT2* transcript levels were compared under diverse abiotic stress conditions in *Physcomitrium patens* and *Spirogyra pratensis*. (A) Expression data for *P. patens* were obtained from the Streptotime web interface (rshiny.gwdg.de/apps/streptotime/; Rieseberg et al., 2025) and represent responses to heat, cold, and high light stress. *EGT1* (Pp3c20_13320V3.1) and *EGT2* (Pp3c8_13110V3.1) expression levels are displayed as box plots showing Z-score transformed normalized log2 counts per million (CPM), obtained from the web interface. (B) For *S. pratensis*, *EGT1* (Sp_v1_0036840.1) and *EGT2* (Sp_v1_0018550.1) expression data originate from experiments utilizing temperature and light intensity gradients (peatmoss.plantcode.cup.uni-freiburg.de/easy_gdb/index.php; Goldbecker et al., 2025) and are visualized as heatmaps, where red indicates high expression and yellow indicates low expression.


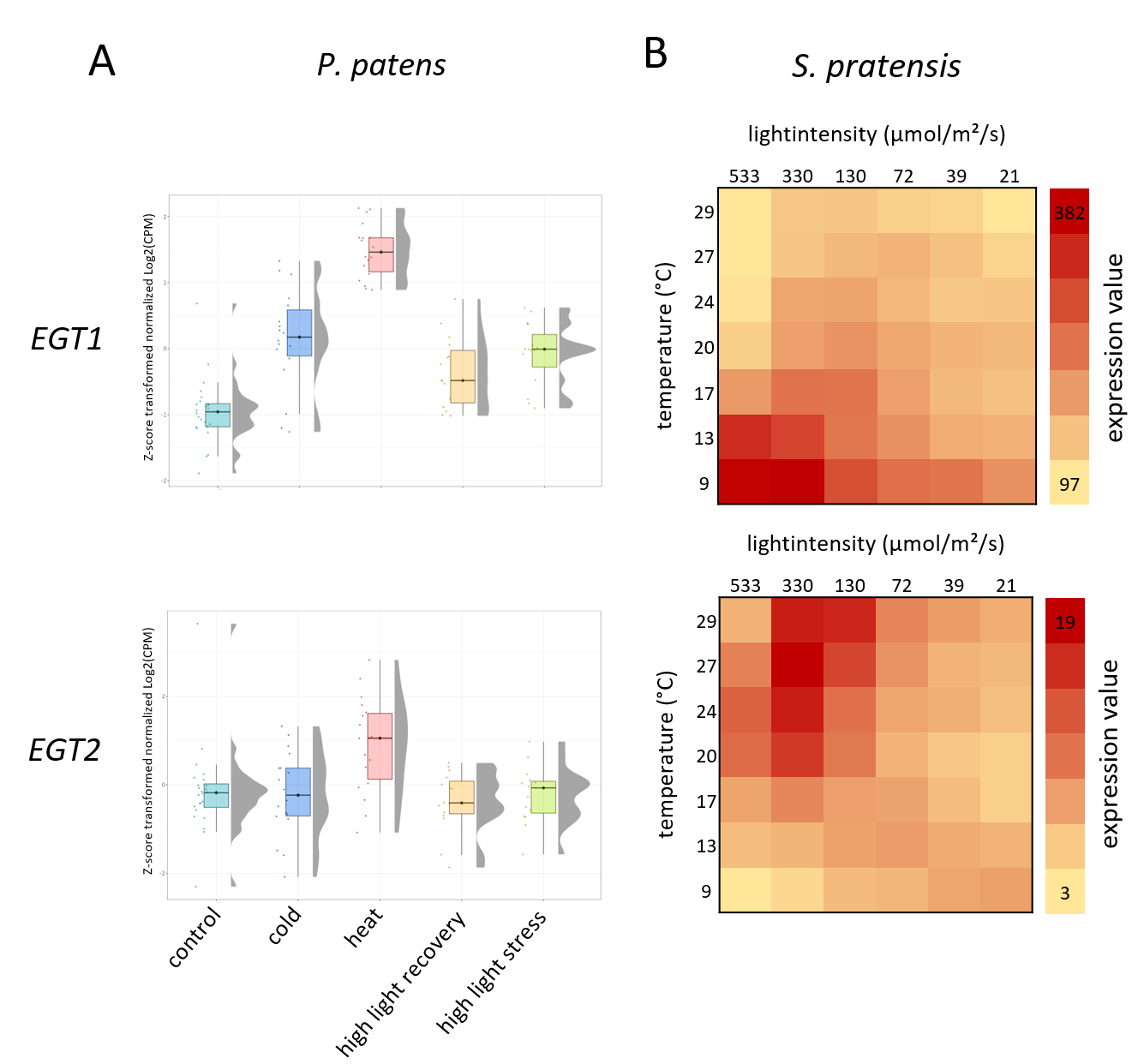


**Supporting Figure 2:** Calibration curve for monobromobimane (mBBr)-EGT quantification. Standards covering 1 – 729 ng mL⁻¹ were prepared. For each standard the mBBr-EGT chromatographic signal was integrated to obtain the peak area, and resulting data were used to generate the calibration equation.


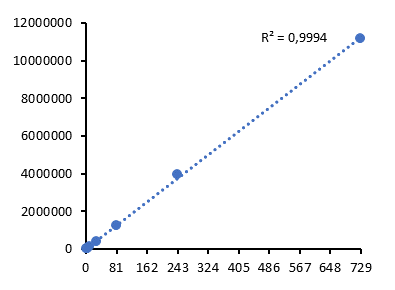


ng mL^-1^ mBBr-EGT

peak area
